# Supplementary material for: The Landscape of lncRNAs in Hepatocellular Carcinoma: A Translational Perspective
Source: Cancers (Basel). 2021 May 28;13(11):2651. doi: 10.3390/cancers13112651 (PMC8197910; doi:10.3390/cancers13112651)
Supplement: Supplementary file 1 [file cancers-13-02651-s001.zip › cancers-1225546-supplementary.pdf]

cancers

MDPI

*Review*

# The Landscape of lncRNAs in Hepatocellular Carcinoma: A Translational Perspective

**Juan Pablo Unfried [1,*,†], Paloma Sangro [2,*,†], Laura Prats-Mari [1], Bruno Sangro [2,3,4,‡] and Puri Fortes [1,3,4,‡],**

[1] Center for Applied Medical Research (CIMA), Department of Gene Therapy and Regulation of Gene Expression, Universidad de Navarra (UNAV), 31008 Pamplona, Spain; lpratsmari@alumni.unav.es (L.P.-M.); pfortes@unav.es (P.F.)
[2] Liver Unit, Clínica Universidad de Navarra (CUN), 31008 Pamplona, Spain; bsangro@unav.es
[3] Navarra Institute for Health Research (IdiSNA), 31008 Pamplona, Spain
[4] Liver and Digestive Diseases Networking Biomedical Research Centre (CIBERehd), 31008 Pamplona, Spain
* Correspondence: junfried@unav.es (J.P.U.); psangro@unav.es (P.S.)
† These authors contributed equally to this work.
‡ These authors contributed equally to this work.

**Citation:** Unfried, J.P.; Sangro, P.; Prats-Mari, L.; Sangro, B.; Fortes, P. The Landscape of lncRNAs in Hepatocellular Carcinoma: A Translational Perspective. *Cancers* **2021**, *13*, 2651. https://doi.org/10.3390/cancers13112651

Academic Editor: Francesco G. Foschi, Andrea Casadei Gardini and Fabio Conti

Received: date
Accepted: date
Published: 28 May 2021

**Publisher's Note:** MDPI stays neutral with regard to jurisdictional claims in published maps and institutional affiliations.

**Copyright:** © 2021 by the authors. Licensee MDPI, Basel, Switzerland. This article is an open access article distributed under the terms and conditions of the Creative Commons Attribution (CC BY) license (http://creativecommons.org/licenses/by/4.0/).

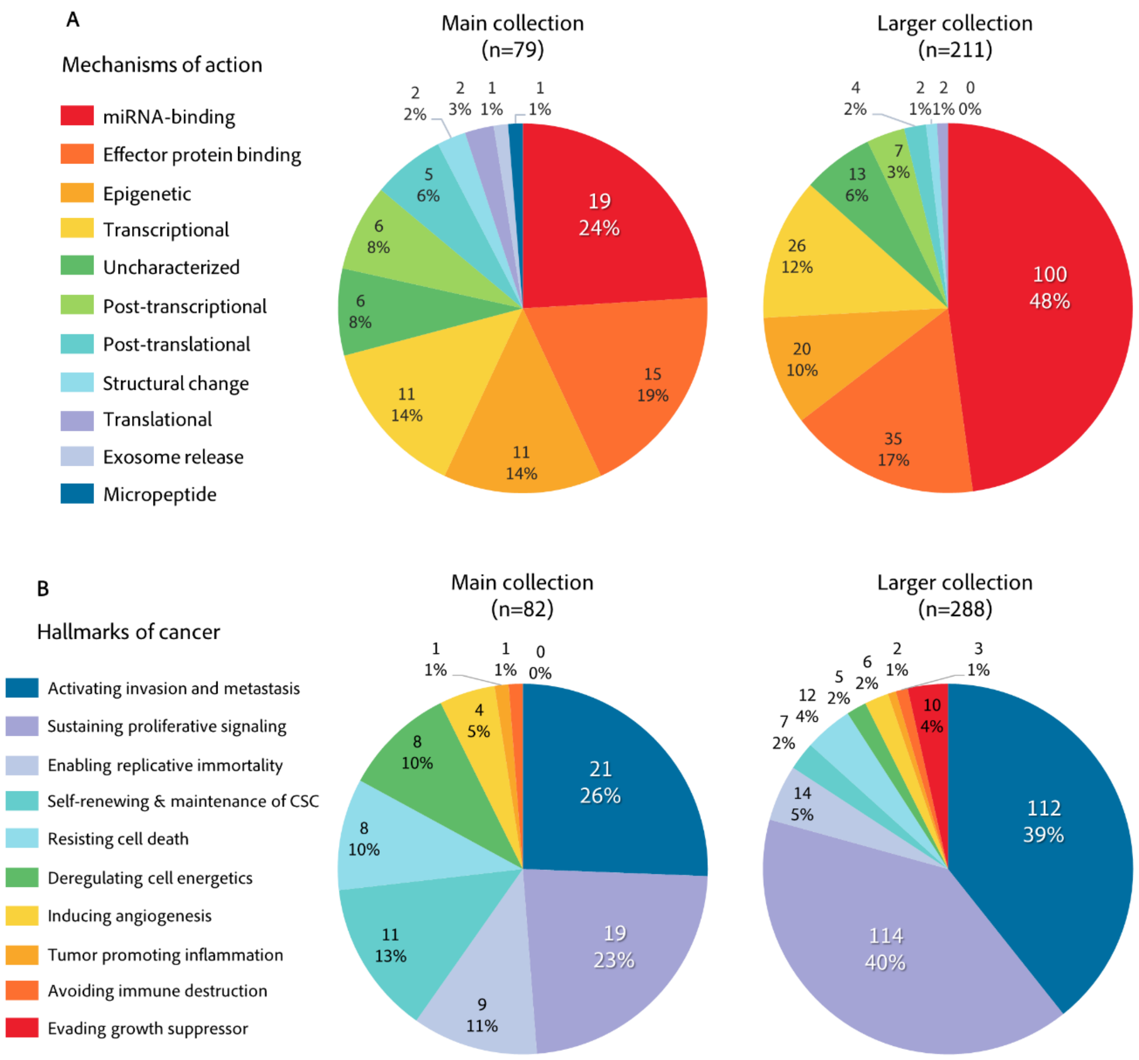


**Figure S1.** Comparison between the lncRNA collection used for the review and a larger collection of HCC-related lncRNAs. (**A**) LncRNAs divided according to their mechanism of action. (**B**) LncRNAs stratified according to the hallmarks of cancer they modulate. Note that a single lncRNA may be ascribed to different mechanisms and/or to several cancer hallmarks. The larger collection comprises all lncRNAs published in journals from the first quartile (Q1) in any possible category up to March 2021. The number of lncRNAs and the percentage is indicated for each case.

**Table S2.** List of lncRNAs included in the larger collection. This list includes the lncRNAs that were used to generate Figure S1, and their annotated mechanisms of action and hallmarks of cancer.

| Number | LncRNA | Hallmark | Mechanism |
|---|---|---|---|
| 1 | LncRNA-ATB | Activating invasion and metastasis | miR-binding |
| 2 | LncRNA-HEIH | Enabling replicative immortality | Epigenetic |
| 3 | MEG3 | Sustaining proliferative signals | miR-binding |
| 4 | PVT1 | Sustaining proliferative signals, Enabling replicative immortality | Binding |
| 5 | HOTTIP | Activating invasion and metastasis | Epigenetic |
| 6 | DANCR | Enabling replicative immortality | Binding |
| 7 | DREH | Activating invasion and metastasis | Binding |
| 8 | H19 | Activating invasion and metastasis, angiogenesis | Binding |
| 9 | CCAT1 | Sustaining proliferative signals, activating invasion and metastasis | miR-binding |
| 10 | ZEB1-AS1 | Activating invasion and metastasis | Transcriptional |
| 11 | HOXD-AS1 | Activating invasion and metastasis | miR-binding |
| 12 | LincRNA-UFC1 | Sustaining proliferative signals | Binding |
| 13 | DILC | Sustaining proliferative signals, enabling replicative immortality | Transcriptional |
| 14 | TUG1 | Sustaining proliferative signals | Epigenetic |
| 15 | Linc00152 | Sustaining proliferative signals | miR-binding |
| 16 | LALR1 | Enabling replicative immortality | Binding |
| 17 | ANRIL | Sustaining proliferative signals | Transcriptional |
| 18 | MCM3AP-AS1 | Sustaining proliferative signals and Evading growth suppression | miR-binding |
| 19 | SNHG6-003 | Sustaining proliferative signals | miR-binding |
| 20 | SNHG12 | Sustaining proliferative signals | miR-binding |
| 21 | AFAP1-AS1 | Activating invasion and metastasis and Evading growth suppression | Binding |
| 22 | Unigene56159 | Activating invasion and metastasis | miR-binding |
| 23 | AOC4P | Activating invasion and metastasis | Binding |
| 24 | uc.134 | Sustaining proliferative signals and Activating invasion and metastasis | Binding |
| 25 | UCA1 | Sustaining proliferative signals | Epigenetic |
| 26 | Ftx | Sustaining proliferative signals | miR-binding |
| 27 | URHC | Sustaining proliferative signals and Resisting cell death | Transcriptional |
| 28 | PTTG3P | Sustaining proliferative signals,<br>enabling replicative immortality and activating invasion and metastasis | Binding |
| 29 | CASC9 | Sustaining proliferative signals and resisting cell death | Binding |
| 30 | CDKN2B-AS1 | Sustaining proliferative signals, Activating invasion and metastasis | miR-binding |
| 31 | miR503HG | Activating invasion and metastasis | Binding |
| 32 | HANR | Sustaining proliferative signals | Binding |
| 33 | PANDAR | Inducing angiogenesis, Activating invasion and metastasis | Uncharacterized |
| 34 | HCAL | Sustaining proliferative signals, Activating invasion and metastasis | miR-binding |
| 35 | SNHG3 | Activating invasion and metastasis | miR-binding |
| 36 | SPRY4-IT1 | Sustaining proliferative signals and Activating invasion and metastasis | Epigenetic |
| 37 | SNHG5 | Sustaining proliferative signals | miR-binding |
| 38 | NEAT1 | Sustaining proliferative signals | miR-binding |
| 39 | SNHG20 | Activating invasion and metastasis | Epigenetic |
| 40 | CCHE1 | Sustaining proliferative signals | Uncharacterized |
| 41 | MIR31HG | Sustaining proliferative signals, Activating invasion and metastasis | miR-binding |
| 42 | Linc00210 | Sustaining proliferative signals, enabling replicative immortality | Binding |
| 43 | SNHG8 | Sustaining proliferative signals, Activating invasion and metastasis | miR-binding |
| 44 | ENSG00000258332.1 | Inducing angiogenesis, Activating invasion and metastasis | Uncharacterized |
| 45 | LINC00635 | Activating invasion and metastasis | Uncharacterized |
| 46 | DLX6-AS1 | Sustaining proliferative signals, Activating invasion and metastasis | miR-binding |
| 47 | HOXA-AS2 | Sustaining proliferative signals | Uncharacterized |
| 48 | HOTAIR | Enabling replicative immortality | Binding |
| 49 | uc002mbe.2 | Resisting cell death | Uncharacterized |

| | | | |
|---|---|---|---|
| 50 | LINC00052 | Activating invasion and metastasis | miR-binding |
| 51 | HOXD-AS1 | Activating invasion and metastasis, Resisting cell death | miR-binding |
| 52 | LINC01554 | Deregulating cellular energetics | Post-transcriptional |
| 53 | MIR22HG | Sustaining proliferative signals, Activating invasion and metastasis | miR-binding |
| 54 | AGAP2-AS1 | Sustaining proliferative signals, Activating invasion and metastasis | miR-binding |
| 55 | Linc-ROR | Genome instability and mutations | miR-binding |
| 56 | Linc00176 | Sustaining proliferative signals | miR-binding |
| 57 | LINC00052 | Sustaining proliferative signals, Activating invasion and metastasis | miR-binding |
| 58 | MITA1 | Activating invasion and metastasis | Uncharacterized |
| 59 | KTN1-AS1 | Sustaining proliferative signals | miR-binding |
| 60 | ICR | Enabling replicative immortality | Binding |
| 61 | GHET1 | Sustaining proliferative signals | Epigenetic |
| 62 | CPS1-IT1 | Activating invasion and metastasis | Binding |
| 63 | SNHG1 | Evading growth suppression | Binding |
| 64 | MIAT | Sustaining proliferative signals, Activating invasion and metastasis | miR-binding |
| 65 | LINC00958 | Sustaining proliferative signals | miR-binding |
| 66 | Lnc-UCID | Evading replicative immortality | Binding |
| 67 | NKILA | Sustaining proliferative signals | Post-transcriptional |
| 68 | Linc-ROR | Activating invasion and metastasis | miR-binding |
| 69 | CCAT2 | Sustaining proliferative signals | miR-binding |
| 70 | NEAT1 | Sustaining proliferative signals | miR-binding |
| 71 | NNT-AS1 | Sustaining proliferative signals | miR-binding |
| 72 | GHET1 | Sustaining proliferative signals | Binding |
| 73 | DGCR5 | Sustaining proliferative signals | miR-binding |
| 74 | n335586 | Activating invasion and metastasis | miR-binding |
| 75 | LINC01225 | Sustaining proliferative signals, Activating invasion and metastasis | Binding |
| 76 | LINC01287 | Sustaining proliferative signals | miR-binding |
| 77 | Linc-USP16 | Sustaining proliferative signals, Activating invasion and metastasis | miR-binding |
| 78 | CRNDE | Sustaining proliferative signals | Binding |
| 79 | HNF1A-AS1 | Sustaining proliferative signals, Activating invasion and metastasis | Post-transcriptional |
| 80 | PDIA3P1 | Evading growth suppression | miR-binding |
| 81 | MAGI2-AS3 | Sustaining proliferative signals | miR-binding |
| 82 | CDKN2BAS | Activating invasion and metastasis | miR-binding |
| 83 | PCNAP1 | Enabling replicative immortality | miR-binding |
| 84 | DLX6-AS1 | Sustaining proliferative signals Enabling replicative immortality | Uncharacterized |
| 85 | SOX21-AS1 | Evading growth suppression | Epigenetic |
| 86 | LNCRNA00364 | Sustaining proliferative signals | Post-transcriptional |
| 87 | NEAT1 | Evading growth suppression | miR-binding |
| 88 | PSTAR | Enabling replicative immortality | Epigenetic |
| 89 | MALAT1 | Tumor promoting inflammation | Post-Translational |
| 90 | DBH-AS1 | Sustaining proliferative signals | miR-binding |
| 91 | HOXA-AS2 | Activating invasion and metastasis | miR-binding |
| 92 | PCAT-14 | Angiogenesis, Activating invasion and metastasis | Transcriptional |
| 93 | TPTEP1 | Sustaining proliferative signals | Binding |
| 94 | UBE2CP3 | Activating invasion and metastasis | Transcriptional |
| 95 | FENDRR | Avoiding immune destruction | miR-binding |
| 96 | RUNX1-IT1 | Sustaining proliferative signals | miR-binding |
| 97 | lnc-ATB | Activating invasion and metastasis | Post-translational |
| 98 | H19 | Sustaining proliferative signals, inducing angiogenesis, activating invasion and metastasis | miR-binding |

| | | | |
|---|---|---|---|
| 99 | HNF1A-AS1 | Sustaining proliferative signals | Epigenetic |
| 100 | LINC00662 | Sustaining proliferative signals | miR-binding |
| 101 | HULC | Sustaining proliferative signals, activating invasion and metastasis resisting cell death | miR-binding |
| 102 | IHS | Activating invasion and metastasis | Binding |
| 103 | LINC-ROR | Sustaining proliferative signals | miR-binding |
| 104 | Linc-GALH | Activating invasion and metastasis | Post-translational |
| 105 | FEZF1-AS1 | Activating invasion and metastasis | Uncharacterized |
| 106 | SNHG7 | Sustaining proliferative signals, Activating invasion and metastasis | miR-binding |
| 107 | GAS5 | Activating invasion and metastasis | miR-binding |
| 108 | SVUGP2 | Activating invasion and metastasis | Transcriptional |
| 109 | uc-002 mbe.2 | Evading growth suppression | Binding |
| 110 | lncRNA-ATB | Activating invasion and metastasis | Binding |
| 111 | GABPB1-AS1 | Deregulating cellular energetics | Transcriptional |
| 112 | AY927503 | Activating invasion and metastasis | Transcriptional |
| 113 | LncRNA-6195 | Sustaining proliferative signals | Binding |
| 114 | MALAT1 | Activating invasion and metastasis, sustaining proliferative signals | miR-binding |
| 115 | LINC00707 | Sustaining proliferative signals | miR-binding |
| 116 | SNHG16 | Sustaining proliferative signals, Activating invasion and metastasis | miR-binding |
| 117 | LncDQ | Activating invasion and metastasis | Epigenetic |
| 118 | DLGAP1-AS1 | Sustaining proliferative signals, Activating invasion and metastasis | miR-binding |
| 119 | CARLo-5 | Activating invasion and metastasis | miR-binding |
| 120 | LINC00511 | Sustaining proliferative signals, Activating invasion and metastasis | miR-binding |
| 121 | MAGI2-AS3 | Sustaining proliferative signals, Activating invasion and metastasis | Transcriptional |
| 122 | LINC00460 | Sustaining proliferative signals | miR-bindning |
| 123 | AK002107 | Activating invasion and metastasis | miR-binding |
| 124 | LINC00161 | Activating invasion and metastasis | Uncharacterized |
| 125 | UC001kfo | Activating invasion and metastasis | Binding |
| 126 | LINC00160 | Resisting cell death | miR-binding |
| 127 | MFI2-AS1 | Sustaining proliferative signals, Activating invasion and metastasis | miR-binding |
| 128 | SLNC2A1-AS1 | Deregulating cellular energetics | Transcriptional |
| 129 | TCL6 | Sustaining proliferative signals, Activating invasion and metastasis | miR-binding |
| 130 | LINC00978 | Sustaining proliferative signals, enabling replicative immortality, Activating invasion and metastasis | Epigenetic |
| 131 | CACNA1G-AS1 | Sustaining proliferative signals, Activating invasion and metastasis | miR-binding |
| 132 | DCST1-AS1 | Resisting cell death | miR-binding |
| 133 | lncWDR26 | Sustaining proliferative signals, Activating invasion and metastasis | Transcriptional |
| 134 | FUNDC2P4 | Activating invasion and metastasis | Transcriptional |
| 135 | HOTAIR | Deregulating cellular energetics | miR-binding |
| 136 | LINC00662 | Sustaining proliferative signals | Binding |
| 137 | lnc-ELF209 | Activating invasion and metastasis | Transcriptional |
| 138 | ZFPM2-AS1 | Activating invasion and metastasis | miR-binding |
| 139 | CSMD1-1 | Sustaining proliferative signals, Activating invasion and metastasis | Binding |
| 140 | LINC00346 | Sustaining proliferative signals, Activating invasion and metastasis | miR-binding |
| 141 | MINCR | Sustaining proliferative signals, Activating invasion and metastasis | Uncharacterized |
| 142 | LNC-HC | Sustaining proliferative signals | miR-binding |
| 143 | PITPNA-AS1 | Sustaining proliferative signals | miR-binding |
| 144 | LINC1433 | Sustaining proliferative signals | miR-binding |
| 145 | C1QTNF1-AS1 | Sustaining proliferative signals, activating invasion and metastasis, Resisting cell death | miR-binding |
| 146 | SNAI3-AS1 | Sustaining proliferative signals, Activating invasion and metastasis | miR-binding |
| 147 | ID2-AS1 | Activating invasion and metastasis | Transcriptional |
| 148 | HOXA11-AS | Enabling replicative immortality, Sustaining proliferative signals | Transcriptional |
| 149 | RNF169 | Genome instability and mutation | Binding |

| | | | |
|---|---|---|---|
| 150 | LASP1-AS | Sustaining proliferative signals, Activating invasion and metastasis | Transcriptional |
| 151 | TRERNA1 | Activating invasion and metastasis | Epigenetic |
| 152 | LINC01391 | Sustaining proliferative signals, Activating invasion and metastasis | Binding |
| 153 | ASMTL-AS1 | Sustaining proliferative signals, Activating invasion and metastasis | miR-binding |
| 154 | LINC01278 | Activating invasion and metastasis | miR-binding |
| 155 | LINC01134 | Sustaining proliferative signals, Activating invasion and metastasis | miR-binding |
| 156 | LOC105369748 | Sustaining proliferative signals, Activating invasion and metastasis | miR-binding |
| 157 | LEF1-AS1 | Sustaining proliferative signals, activating invasion and metastasis, Inducing angiogenesis | miR-binding |
| 158 | MYLK-AS1 | Sustaining proliferative signals | Binding |
| 159 | MYLK-AS1 | Sustaining proliferative signals and Inducing angiogenesis | miR-binding |
| 160 | lnc-lp53 | Sustaining proliferative signals, genome stability and mutations | Binding |
| 161 | LINC01352 | Sustaining proliferative signals, Activating invasion and metastasis | miR-binding |
| 162 | LINC01149 | Avoiding immune destruction | miR-binding |
| 163 | EVA1A-AS | Sustaining proliferative signals | Transcriptional |
| 164 | LINC00961 | Sustaining proliferative signals, Activating invasion and metastasis | miR-binding |
| 165 | LINC00624 | Sustaining proliferative signals, Activating invasion and metastasis | Binding |
| 166 | LL22NC03-N14H11.1 | Sustaining proliferative signals, activating invasion and metastasis, deregulating cellular energetics | Transcriptional |
| 167 | RP11-295G20.2 | Sustaining proliferative signals, activating invasion and metastasis resisting cell death | miR-binding |
| 168 | LINC01419 | Sustaining proliferative signals, Activating invasion and metastasis | Transcriptional |
| 169 | LINC01419 | Sustaining proliferative signals, Activating invasion and metastasis | Epigenetic |
| 170 | LINC01123 | Sustaining proliferative signals, Activating invasion and metastasis | miR-binding |
| 171 | LINC00628 | Activating invasion and metastasis | Transcriptional |
| 172 | DNM3OS | Sustaining proliferative signals, Activating invasion and metastasis | Binding |
| 173 | SNHG6 | Evading growth suppression | miR-Binding |
| 174 | RP11-286H15.1 | Sustaining proliferative signals | Binding |
| 175 | MAPKAPK5-AS1 | Sustaining proliferative signals, Activating invasion and metastasis | miR-binding |
| 176 | CCDC183-AS1 | Sustaining proliferative signals, Activating invasion and metastasis | miR-binding |
| 177 | Lnc-APUE | Cell cycle progression, Sustaining proliferative signals | miR-binding |
| 178 | LINC00680 | Enabling replicative immortality, Evading growth suppression | miR-binding |
| 179 | SNHG14 | Sustaining proliferative signals, Activating invasion and metastasis | miR-binding |
| 180 | Linc-SCRG1 | Sustaining proliferative signals, Activating invasion and metastasis | miR-binding |
| 181 | ZFPM2-AS1 | Evading growth suppression, sustaining proliferative signals, activating invasion and metastasis | miR-binding |
| 182 | Linc00261 | Activating invasion and metastasis | Transcription |
| 183 | Linc-KILH | Sustaining proliferative signals | Binding |
| 184 | LINC-PINT | Resisting cell death, Sustaining proliferative signals | Micropeptide |
| 185 | linc-FAM138B | Sustaining proliferative signals, Activating invasion and metastasis | miR-binding |
| 186 | UPK1A-AS1 | Sustaining proliferative signals | Epigenetic |
| 187 | LINC01134 | Activating invasion and metastasis | Transcriptional |
| 188 | MACC1-AS1 | Sustaining proliferative signals, Activating invasion and metastasis | Transcriptional |
| 189 | DLEU2 | Enabling replicative immortality | Epigenetic |
| 190 | RERT | Enabling replicative immortality | Structural change |
| 191 | MUF | Activating invasion and metastasis | miR-binding |
| 192 | GATA3-AS | Activating invasion and metastasis | Post-transcriptional |
| 193 | lncMER52A | Activating invasion and metastasis | Post-translational |
| 194 | HAND2-AS1 | Activating invasion and metastasis | Epigenetic |
| 195 | HCCL5 | Activating invasion and metastasis | Uncharacterized |
| 196 | CASC2 | Activating invasion and metastasis | miR-binding |
| 197 | PTENP1 | Activating invasion and metastasis | miR-binding |
| 198 | SNHG10 | Activating invasion and metastasis | miR-binding |

| | | | |
|---|---|---|---|
| 199 | ZFAS1 | Activating invasion and metastasis | miR-binding |
| 200 | Lnc-EGFR | Avoiding immune destruction | Binding |
| 201 | LINC00665 | Tumor promoting inflammation | Post translational |
| 202 | MVIH | Inducing angiogenesis | Uncharacterized |
| 203 | HOTTIP | Resisting cell death | Epigenetic |
| 204 | PRAL | Resisting cell death | Structural change |
| 205 | LncBRM | Enabling replicative immortality | Binding |
| 206 | Lnc-B-Catm | Enabling replicative immortality | Epigenetic |
| 207 | LncTCF7 | Enabling replicative immortality | Epigenetic |
| 208 | LncSox4 | Enabling replicative immortality | Transcriptional |
| 209 | PXN-AS1 | Sustaining proliferative signals | Translational |
| 210 | LINC00998 | Sustaining proliferative signals | Micropeptide |
